# Visual tests predict dementia risk in Parkinson disease

**DOI:** 10.1212/CPJ.0000000000000719

**Published:** 2020-02

**Authors:** Louise-Ann Leyland, Fion D. Bremner, Ribeya Mahmood, Sam Hewitt, Marion Durteste, Molly R.E. Cartlidge, Michelle M.-M. Lai, Luke E. Miller, Ayse P. Saygin, Pearse A. Keane, Anette E. Schrag, Rimona S. Weil

**Affiliations:** Dementia Research Centre (L-AL, RM, RSW), Institute of Neurology, University College London, United Kingdom; Neuro-ophthalmology (FDB, MM-ML), National Hospital for Neurology and Neurosurgery, University College London Hospitals, London, United Kingdom; Institute of Neurology (SH, MD, MREC), University College London, UCL, United Kingdom; School of Biomedical Sciences (MREC), Biological Sciences, Leeds University, United Kingdom; ImpAct (LEM), Lyon Neuroscience Research Center, France; Department of Cognitive Science (APS), University of California, San Diego; Kavli Institute for Brain and Mind (APS), University of California, San Diego; Institute of Ophthalmology (PAK), UCL, United Kingdom; Moorfields Eye Hospital (PAK), London, United Kingdom; Department of Clinical Neuroscience (AES), Institute of Neurology, UCL Hampstead Campus, London, United Kingdom; Movement Disorders Consortium (AES, RSW), UCL, United Kingdom; and The Wellcome Centre for Human Neuroimaging (RSW), Institute of Neurology, University College London, United Kingdom.

## Abstract

**Objective:**

To assess the role of visual measures and retinal volume to predict the risk of Parkinson disease (PD) dementia.

**Methods:**

In this cohort study, we collected visual, cognitive, and motor data in people with PD. Participants underwent ophthalmic examination, retinal imaging using optical coherence tomography, and visual assessment including acuity and contrast sensitivity and high-level visuoperception measures of skew tolerance and biological motion. We assessed the risk of PD dementia using a recently described algorithm that combines age at onset, sex, depression, motor scores, and baseline cognition.

**Results:**

One hundred forty-six people were included in the study (112 with PD and 34 age-matched controls). The mean disease duration was 4.1 (±2·5) years. None of these participants had dementia. Higher risk of dementia was associated with poorer performance in visual measures (acuity: ρ = 0.29, *p* = 0.0024; contrast sensitivity: ρ = −0.37, *p* < 0.0001; skew tolerance: ρ = −0.25, *p* = 0.0073; and biological motion: ρ = −0.26, *p* = 0.0054). In addition, higher risk of PD dementia was associated with thinner retinal structure in layers containing dopaminergic cells, measured as ganglion cell layer (GCL) and inner plexiform layer (IPL) thinning (ρ = −0.29, *p* = 0.0021; ρ = −0.33, *p* = 0.00044). These relationships were not seen for the retinal nerve fiber layer that does not contain dopaminergic cells and were not seen in unaffected controls.

**Conclusion:**

Visual measures and retinal structure in dopaminergic layers were related to risk of PD dementia. Our findings suggest that visual measures and retinal GCL and IPL volumes may be useful to predict the risk of dementia in PD.


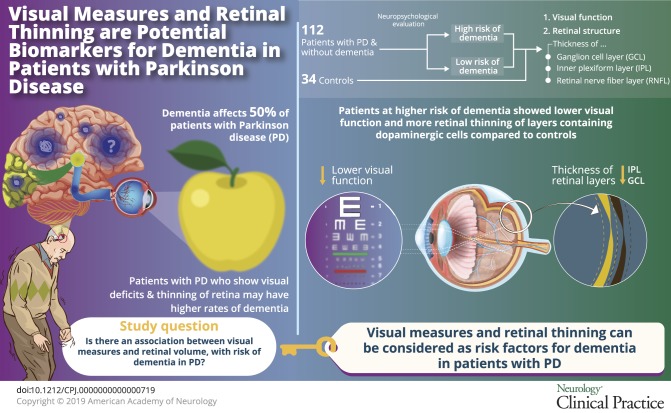


Dementia is a debilitating aspect of Parkinson disease (PD), affecting 50% of patients within 10 years of diagnosis, with variability in timing and severity. Patients with PD who show visual deficits including color and higher-order visual changes may have higher rates of converting to PD dementia or develop dementia earlier in their disease course.^1–3^ However, this has not yet been examined systematically, and whether earlier stages of visual processing and retinal structure are linked to the risk of PD dementia is not yet known.

Retinal structure can be imaged noninvasively using optical coherence tomography (OCT)^4^ and shows thinning in Alzheimer disease^5^ and as a population screen for dementia.^6–8^ Retinal structural changes are seen in PD, but early studies of retinal nerve fiber layer (RNFL) thinning in PD were not replicated.^9^ The location of dopaminergic amacrine cells in the inner plexiform layer (IPL)^10^ in nonhuman studies, and postmortem findings of phosphorylated alpha-synuclein in the IPL, suggest that deeper layers are more likely to be affected in PD.^11^ Recent studies show consistent thinning in the IPL and ganglion cell layer (GCL) in PD,^12^ but whether IPL or GCL thinning is linked to PD dementia is not known.

New algorithms have emerged that combine measures such as age and motor severity with sensitivity to predict cognitive change in PD.^13–15^ Although these algorithms are useful, they do not allow tracking of disease progression or relate directly to changes in the parkinsonian brain.

We therefore examined the association between visual measures and retinal structure, with the risk of dementia in PD. These measures have potential for stratifying high-risk patients for clinical trials.

## Methods

### Participants

One hundred seventeen people with PD were recruited from our UK center between October 2017 and November 2018. Inclusion criteria were early-stage PD (Queen Square Brain Bank criteria), within 10 years of diagnosis, aged 49–82 years. Exclusion criteria were confounding neurologic or psychiatric disorders, a diagnosis of dementia or Mini-Mental State Examination (MMSE) less than 25,^13^ and ophthalmic disease sufficient to impair visual acuity. Two patients were excluded because of dementia, and 3 were excluded because of ophthalmic disease (glaucoma). Therefore, the data reported here include 112 people with PD. Thirty-five age-matched controls unaffected by neurologic, psychiatric, or ophthalmic disease were additionally recruited from university databases and unaffected spouses. Of these, 1 was excluded because of developing mild cognitive impairment within 6 months of taking part, leaving 34 controls in the analysis reported here.

### Clinical and ophthalmic evaluation

All participants were tested on their usual medications and levodopa equivalent daily dose calculated. Symptom severity was assessed using the Movement Disorders Society Unified PD Rating Scale (UPDRS). Olfaction was assessed using the odor identification subset of the Sniffin' sticks test. Participants completed the Hospital Anxiety and Depression Scale and REM Sleep Behavior Disorder Screening Questionnaire (RBDSQ). A comprehensive ophthalmic assessment was performed by a consultant ophthalmologist. This included slit-lamp ophthalmic examination and measurement of intraocular pressures using Goldmann applanation tonometry.

### Assessments of visual function

**Figure 1:**
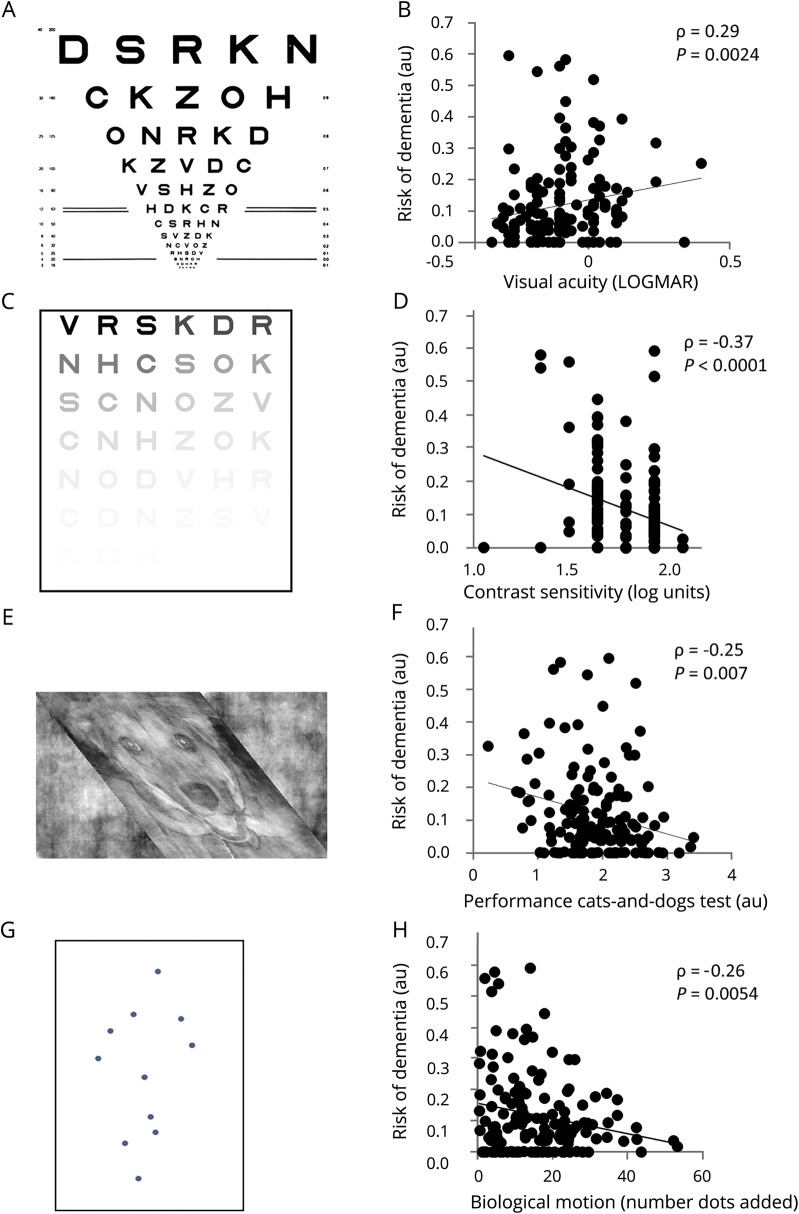
Tests of visual function and relationship with risk of Parkinson disease (PD) dementia (A) LogMAR visual acuity chart. Adapted by permission from BMJ Publishing Group Limited.^35^ (B) Relationship between the risk of PD dementia and visual acuity. (C) Pelli-Robson chart for assessing contrast sensitivity. (D) Relationship between the risk of PD dementia and contrast sensitivity. (E) Cats-and-Dogs test of higher-order visuoperception. The task is to identify whether the animal shown is a cat or a dog, with differing amounts of skew applied to the image to determine the level of skew tolerated. (F) Relationship between the risk of PD dementia and higher-order visuoperception, tested by skew tolerance. (G) Biological motion. Dots are shown at the position of the major joints of the body. The dots move to give the strong percept of a person walking. Extra dots are added, and the number of dots tolerated, where the participant can still detect a person moving, is calculated. (H) Relationship between the risk of PD dementia and higher-order visuoperception, tested with biological motion. Poorer performance in each of these measures is linked with a higher risk of PD dementia.

Visual measures (figure 1) were all performed before mydriasis. Visual acuity was measured binocularly using a logMAR chart (with eyeglasses, if worn) (figure 1A). Contrast sensitivity was measured binocularly (with eyeglasses, if worn) using a Pelli-Robson chart (SSV-281-PC) (sussex-vision.co.uk) (figure 1C). Color vision was assessed using the D15 test, and error scores log transformed.

Visuoperception was measured using 2 tasks probing distinct aspects of higher-order visuoperception: the Cats-and-Dogs test measures tolerance to visual skew (figure 1E) and has been associated with PD.^16^ Stimuli were generated as previously described.^2,16^ Images were presented centrally, subtending 4° × 13° of visual angle and shown for 280 ms, followed by a choice screen (response time 3,800 ms), 90 repetitions (total time 15 minutes). To calculate discrimination sensitivity, missing trials were excluded and performance at each level of skew calculated. A sigmoid psychometric curve was fitted, and threshold for image detection was calculated at 75% performance. Biological motion measures sensitivity to detect the appearance of a moving person from moving dots at the position of the major joints of a person.^17^ It is known to be affected in PD.^18^ Stimuli consisted of point-light walkers (12 white dots on gray background, height 7°) (figure 1G). Control stimuli were generated using point-light walkers with dot position and motion scrambled. Motion-matched noise dots were added to increase difficulty, using an adaptive staircase procedure.^17^ Stimuli were presented for 800 ms. Participants determined whether the animation depicted a moving person or scrambled moving dots, 225 repetitions, total time 15 minutes. We used the QUEST Bayesian adaptive method to calculate the maximum number of additional noise dots tolerated for performance to reach 82% accuracy. Stimuli were generated within MATLAB Psychophysics Toolbox 3 implemented on a Dell Latitude 3340 in a darkened room. Whether this test relates to the risk of PD dementia is not known.

### Retinal structure: OCT

**Figure 2:**
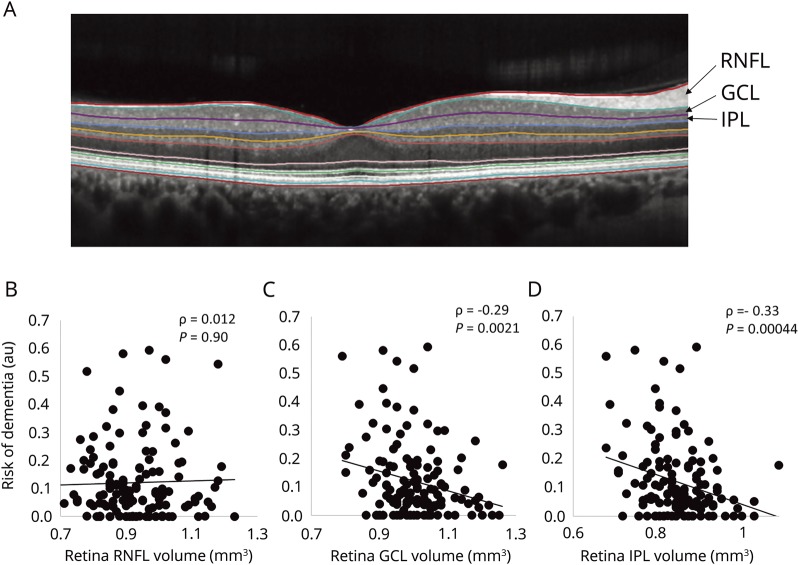
Relationship between the retinal volume and risk of Parkinson disease (PD) dementia (A) Output of optical coherence tomography retinal imaging, with cross section at the macula. Retinal layers identified by automatic segmentation are shown. (B) Relationship between the risk of PD dementia and RNFL volume. (C) Relationship between the risk of PD dementia and GCL volume. (D) Relationship between the risk of PD dementia and IPL volume. Retinal layers that contain dopaminergic cells (GCL and IPL) show greater thinning linked with PD dementia risk. This relationship is not seen in the RNFL that does not contain dopaminergic cells. RNFL = retinal nerve fiber layer; GCL = ganglion cell layer; IPL = inner plexiform layer.

Retinal imaging was performed on both eyes of each participant on the same day as other measures were obtained. Inner retinal layer structure was measured using high-resolution spectral domain optical coherence tomography (SD-OCT; Heidelberg HRA/Spectralis manufactured in 2011) in a dimly lit room after pharmacologic mydriasis according to a standard protocol.^19^ Two OCT devices were used at 1 site (National Hospital for Neurology and Neurosurgery) with 4 operators (L.-A.L., R.M., S.H., and M.R.E.C.), with viewing module v. 6.9.5.0. All participants underwent macular scan protocols in Infrared-OCT mode; laser illumination used for excitation was 486 nm. This allows simultaneous acquisition of a fundus image, with a reflectance wavelength of 816 nm and OCT scan with TruTrack eye-tracking technology to stabilize the retinal image. The volumetric macular protocol of the Spectralis SD-OCT device (NSite application) was used to measure macular thickness and volume (figure 2A). This uses an internal fixation source and centers on the participant's fovea. The protocol consists of 25 vertical line scans at a resolution of 1,536 (scanning angle: 20°×20°, density: 240 μm, 4.7 scans/s, automatic real-time frames: 49). Quality control of OCT data was performed in compliance with international consensus OSCAR 1B OCT quality control standards (OSCAR-IB),^20^ and OCT data were collected and reported according to international APOSTEL guidelines.^21^

### OCT analysis

Automatic layer segmentation was applied, via Heidelberg software v.1.10.20, with Nsite module included, to compute the thickness of the GCL, IPL, and RNFL. Blinded assessors (S.H. and M.R.E.C.) verified layer position, and manual segmentation was undertaken when automatic segmentation deviated from the visible gradient change for that layer (quality checked by L.L.). All layers were segmented and exported, and total volume and total thickness within each of the RNFL, GCL, and IPL were calculated across all 4 quadrants and summed for a total layer thickness, exported as a 1-, 3-, 6-mm EDTRS grid. Retinal data from 9 individuals were excluded because of ophthalmic disease or poor-quality scans (table e-1, links.lww.com/CPJ/A126). One eye from each participant was selected for analysis. In patients with PD, this was the eye contralateral to the most symptomatic side as identified during UPDRS-III. For controls and patients with symmetrical motor signs, the selected eye was selected randomly. In 8 patients and in 1 control, the selected eye could not be used because of ophthalmic disease exclusions and the other eye was included for these participants (table e-1).

### Neuropsychological evaluation

Cognitive assessment was in line with recent Movement Disorder Society guidelines,^22^ with 2 assessments per cognitive domain. General cognitive function was assessed with the MMSE and Montreal Cognitive Assessment. Memory was assessed with the Recognition Memory Test for words and immediate and delayed versions of the Logical Memory task (Wechsler Memory Scale IV). Language was assessed with the Graded Naming Test and letter fluency. Visuospatial abilities were tested with Benton Judgment of Line Orientation and Hooper Visual Organization Test. Executive functions were measured with the Stroop task from the Delis-Kaplan Executive Function System and category fluency. Attention was tested using color naming from the Stroop and Digit Span.

### Defining dementia risk status

We defined dementia risk using a recently described, prospectively validated algorithm.^13^ This combines clinical information on sex, age at disease onset, years of education, UPDRS-III (motor examination), and MMSE to generate a risk score (e-Methods for details, links.lww.com/CPJ/A137). In addition to the continuous scores, we categorized patients with PD into high vs low risk of dementia using a median split of these algorithmic scores. As this algorithm includes age at diagnosis, it cannot be used to stratify controls. We repeated our analysis with 2 other recently described algorithms: one using age, years of education, UPDRS-III, RBDSQ, depression, and olfaction^14^ and the other using age, UPDRS axial scores, and animal fluency^15^ (e-Methods for further details).

### Statistical analyses

Performance was compared between groups using 2-tailed Welch *t* tests or Mann-Whitney-Wilcoxon tests for non-normally distributed data. We used linear regression to examine the effects of visual measures and retinal structure on dementia risk in PD and controls and Spearman rank correlation where data were not normally distributed. *p* < 0.05, Bonferroni corrected for multiple comparisons (8 comparisons, significance <0.0063), was accepted as the threshold for statistical significance. Sample sizes were based on power analyses performed before data collection. Analyses were performed in R (r-project.org/). Data were inspected and outliers (beyond mean ± 3 × SD) removed. Participants with missing data were omitted for that measure (table e-1, links.lww.com/CPJ/A126).

### Standard protocol approvals, registrations, and patient consents

All participants gave written informed consent, and the study was approved by the Queen Square Research Ethics Committee (15.LO.0476).

### Data availability

Anonymized data can be made available by request from qualified investigators to the senior author of this publication.

## Results

### Demographics

One hundred twelve people with PD and 34 unaffected controls took part. Age and sex did not differ between PD and controls: mean age PD 64.3 ± 8 years, mean age controls 64.8 ± 9 years (*t*(49) = 0.3, *p* = 0.74). Fifty-six people with PD were assigned low risk, based on a median split of the risk score,^13^ and 56 were high risk. The average disease duration was 4.1 ± 2.5 years and did not differ between high- and low-risk patients. There was also no difference in motor, sleep, olfaction, or levodopa dose between high- vs low-risk patients (table 1) (age differed between high- and low-risk groups, as this was strongly linked to age at disease onset, that defined risk, and sex was included in the risk algorithm).^13^

**Table 1:**
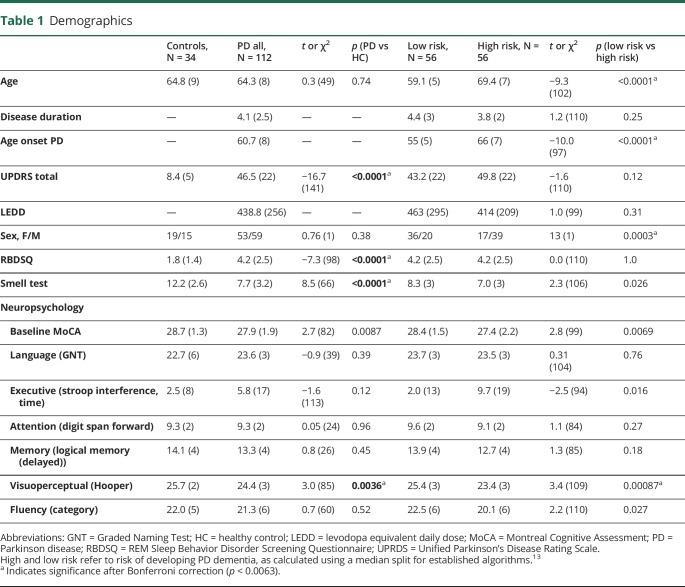
Demographics

### Relationship between visual measures and PD dementia risk

There was no difference in visual measures between people with PD and unaffected controls, apart from poorer higher visual function (Cats-and-Dogs test) in people with PD that did not survive correction for multiple comparisons (table 2). However, when patients were split into high vs low risk of PD dementia using a median split, high-risk patients showed worse performance in almost all visual measures (table 2), with similar differences seen when patients were divided into quartiles for risk (table e-2, links.lww.com/CPJ/A126).

**Table 2:**
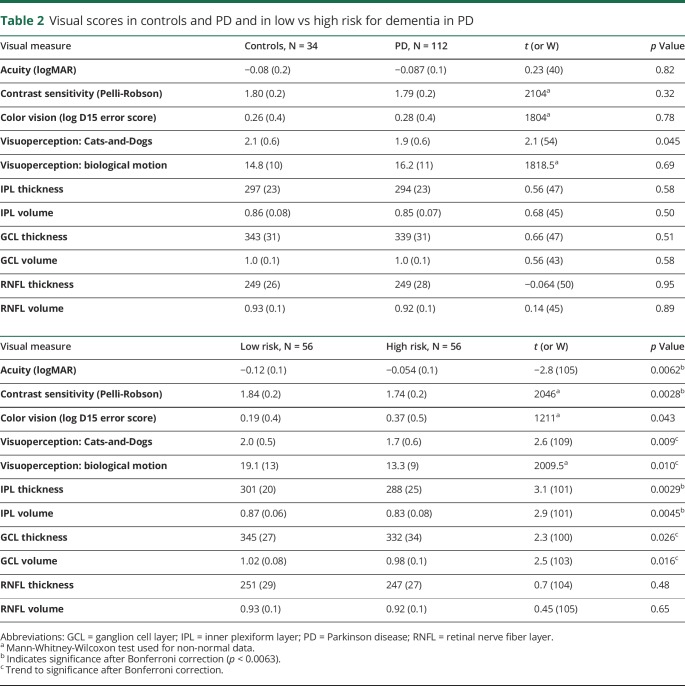
Visual scores in controls and PD and in low vs high risk for dementia in PD

Poorer visual function across several levels of visual processing was associated with a higher risk of dementia (table 3), although patients did not report deficits in visual function. Visual acuity, measured using the logMAR, and contrast sensitivity, measured using the Pelli-Robson and color vision, were all associated with a higher risk of dementia (ρ = 0.29, *p* = 0.0024; ρ = −0.37, *p* < 0.0001; ρ = 0.26, *p* = 0.0054) (table 3 and figure 1A–D). Higher-order visuoperception, measured using biological motion, showed impaired performance in higher-risk patients (ρ = −0.026, *p* = 0.0054), with trend to significance for visual skew (ρ = −0.25, *p* = 0.0073) (table 3 and figure 1E–F).

**Table 3:**
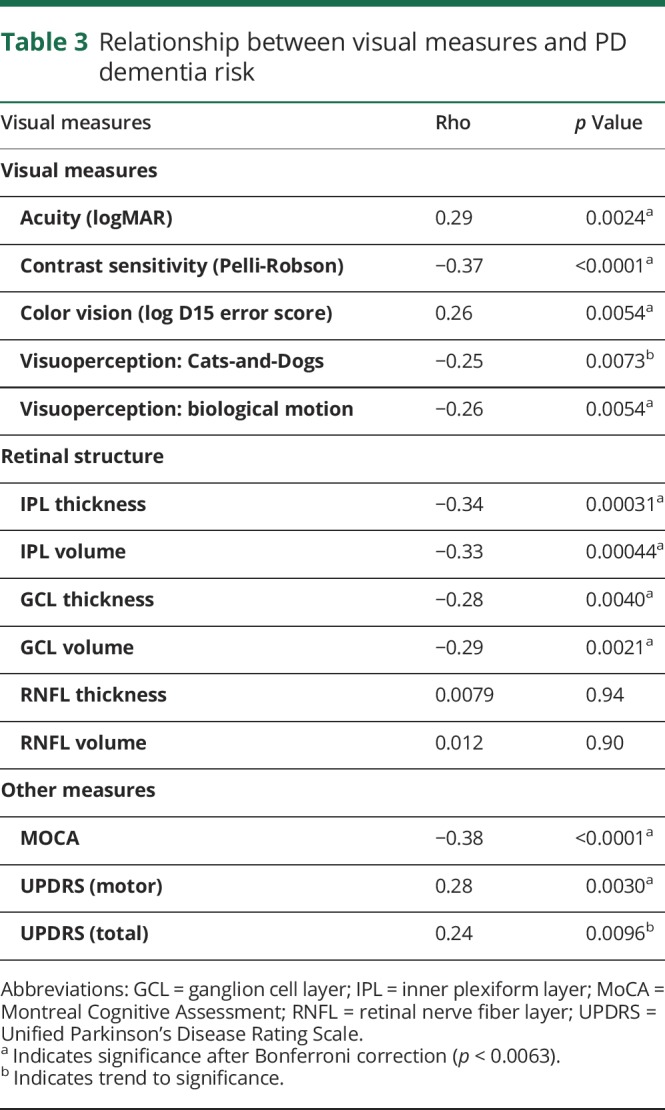
Relationship between visual measures and PD dementia risk

### Relationship between structural retinal measures and PD dementia risk

Patients at a higher risk of dementia showed more retinal thinning in dopamine-containing layers: GCL (ρ = −0.29, *p* = 0.0021) and IPL (ρ = −0.33, *p* = 0.00044). These differences were not seen in the RNFL that does not contain dopaminergic cells (ρ = 0.012, *p* = 0.90 (table 3 and figure 2).

### Generalizability across other estimates of risk

To ensure that our findings were generalizable across different measures of risk, we repeated our analysis using 2 alternative Parkinson dementia risk algorithms, one adapted for clinical values^14^ and found qualitatively the same relationships (table 4).

**Table 4:**
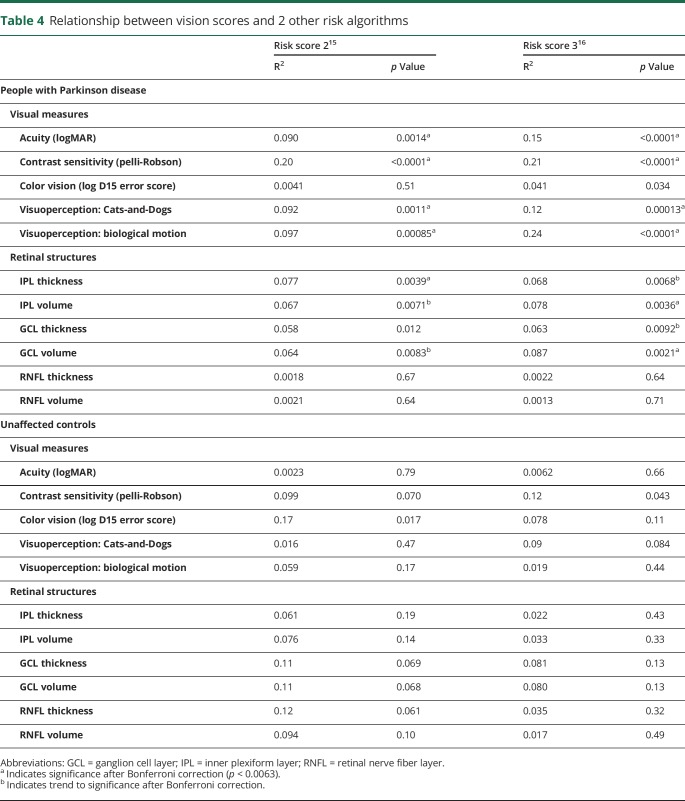
Relationship between vision scores and 2 other risk algorithms

### Relationship between vision and dementia risk in unaffected controls

To test whether these findings were related to the risk of PD dementia, rather than nonspecific effects of aging, we examined these relationships in unaffected controls. We found that unlike in PD, in controls, visual measures were not related to dementia risk scores or age (table 4).

## Discussion

In this large PD cohort, visual measures across multiple stages of visual processing and structural retinal changes are associated with a higher risk of more rapid development of PD dementia.

Isolated visual measures such as pentagon copying and color vision have previously been linked with cognitive changes in PD,^1,3^ and involvement of visual processing brain regions is associated with more rapid PD dementia.^23^ Here, we show that vision along the entire visual processing axis is linked with the risk of cognitive change in PD. This spans from higher-order processes involving posterior brain regions to contrast sensitivity and visual acuity mediated by ophthalmic structures or primary visual cortex and dopamine-containing layers of the retina.

Our finding that retinal thinning is linked with a higher risk of more rapid PD dementia is consistent with a growing literature showing retinal involvement in PD. Dopamine is a key modulatory neurotransmitter in the retina.^24^ Reduced dopamine innervation is seen around the fovea in PD,^25^ and lower retinal dopaminergic concentrations are found at postmortem in untreated PD.^26^ OCT initially suggested RNFL thinning in PD,^9^ but this was not replicated by others^27^ potentially due to methodological differences including sample characteristics, lack of appropriate statistical correction, and segmentation protocols.

As OCT technology has improved, it has become clearer that retinal thinning in PD is restricted to the dopamine-containing layers, the GCL and IPL, rather than the RNFL.^12^ Nuclei of dopaminergic amacrine interneurons lie adjacent to the IPL,^24^ with axons running horizontally across the IPL and GCL,^28^ and postmortem studies show that retinal alpha-synuclein accumulates at the interface with the IPL in PD.^11^ Our finding of a link between retinal thinning and dementia risk in these specific layers has important mechanistic implications for progression of PD dementia.

In the wider population, RNFL thinning is linked with higher rates of cognitive decline,^7^ and another study found that GC-IPL thinning (but not RNFL) is linked with prevalent dementia.^8^ Our finding of a link between retinal thinning and risk of dementia may not be wholly specific to Parkinson dementia and may also apply to other types of dementia. Ultimately, this will need to be tested in prospectively followed cohorts developing different forms of dementia. In PD dementia, RNFL thinning has previously been shown to correlate with MMSE scores,^29^ but this relationship with cognition has not yet been shown in PD without dementia, but at risk of dementia. Recent postmortem findings of retinal phosphorylated alpha-synuclein strengthen the link between the retina and brain disease in PD, as the amount of phosphorylated alpha-synuclein in the retina correlates with the density of Lewy-type alpha-synuclein in the brain.^30^ This anatomic finding supports the use of retinal structural measures as a window into brain pathology.

The reason for selective vulnerability of dopamine-containing GC and IP layers is not known, but may relate to common properties of dopamine-containing cells. These show autonomous spiking behavior with low intrinsic Ca^2+^ buffering.^31^ This may lead to free-radical accumulation and increased vulnerability to neurodegeneration. Whether retinal layers are affected before cortical regions in PD dementia can be examined in longitudinal evaluation.

We did not find impairment of visual dysfunction in the overall PD group compared with controls, apart from skew tolerance (Cats-and-Dogs test); differences were only seen between the high- and low-risk individuals. Previous studies have reported visual dysfunction in PD compared with controls.^2,32,33^ It is therefore possible that previous studies reporting visual deficits in PD overall included higher proportions of high-risk individuals or patients with cognitive involvement.

### Limitations

Although our study examines risk based on cross-sectional data, the algorithms we use are validated using prospective follow-up. Ultimately, however, whether measures identified here truly predict the development of dementia in PD will need to be tested in longitudinal analyses.

A further question is whether retinal and visual measures are affected by the same factors that promote Parkinson dementia, such as age. The relationship between older age and poorer vision is well established,^34^ and higher age at onset and age in itself is strongly linked with development of dementia in PD.^14,15^ Whether some factor such as increased amyloid deposition in cortical and retinal structures is responsible for both effects or whether there are separate processes affecting vision more selectively is not yet known. Age (or age at onset) is incorporated into all algorithmic risk scores. For this reason, we were unable to correct for age in our regression analyses. However, lack of association between visual measures and dementia risk in unaffected controls suggests that this relationship between vision and dementia is more specific to the risk of PD dementia and is not purely a result of deteriorating function with age (although we note that the control group is smaller than the group with PD). We will require longitudinal assessment of visual and cognitive factors in patients with PD to determine and validate whether visual measures such as those presented here show additional sensitivity to recently defined algorithms that use more general clinical inputs.

Our finding of a link between acuity and PD dementia risk raises the question of whether higher-order visual changes are a result of lower acuity or arise independently due to cortical changes in at-risk individuals. This will need to be tested by examining cortical differences in these groups and their timing relative to retinal and acuity deficits.

We show that visual measures and retinal thinning are linked with a higher risk of more rapid PD dementia. This provides useful insights into the development of PD dementia, implicating visual brain regions as being involved at early stages. As Parkinson dementia is becoming better understood, noninvasive visual measures such as those used in this study may have potential to be used alone or in combination with other clinical factors as possible biomarkers for PD dementia and to stratify high-risk patients for disease-modifying trials. This could enable better-powered trials and ultimately may pave the way toward new treatments for PD dementia.
